# Acute Thermal Stressor Increases Glucocorticoid Response but Minimizes Testosterone and Locomotor Performance in the Cane Toad (*Rhinella marina*)

**DOI:** 10.1371/journal.pone.0092090

**Published:** 2014-03-18

**Authors:** Edward J. Narayan, Jean-Marc Hero

**Affiliations:** Environmental Futures Research Institute, School of Environment, Griffith University, Gold Coast Campus, Australia; Leiden University Medical Centre, Netherlands

## Abstract

Climatic warming is a global problem and acute thermal stressor in particular could be considered as a major stressor for wildlife. Cane toads (*Rhinella marina*) have expanded their range into warmer regions of Australia and they provide a suitable model species to study the sub-lethal impacts of thermal stressor on the endocrine physiology of amphibians. Presently, there is no information to show that exposure to an acute thermal stressor could initiate a physiological stress (glucocorticoid) response and secondly, the possible effects on reproductive hormones and performance. Answering these questions is important for understanding the impacts of extreme temperature on amphibians. In this study, we experimented on cane toads from Queensland, Australia by acclimating them to mildly warm temperature (25°C) and then exposing to acute temperature treatments of 30°, 35° or 40°C (hypothetical acute thermal stressors). We measured acute changes in the stress hormone corticosterone and the reproductive hormone testosterone using standard capture and handling protocol and quantified the metabolites of both hormones non-invasively using urinary enzyme-immunoassays. Furthermore, we measured performance trait (i.e. righting response score) in the control acclimated and the three treatment groups. Corticosterone stress responses increased in all toads during exposure to an acute thermal stressor. Furthermore, exposure to a thermal stressor also decreased testosterone levels in all toads. The duration of the righting response (seconds) was longer for toads that were exposed to 40°C than to 30°, 35° or 25°C. The increased corticosterone stress response with increased intensity of the acute thermal stressor suggests that the toads perceived this treatment as a stressor. Furthermore, the results also highlight a potential trade-off with performance and reproductive hormones. Ultimately, exposure acute thermal stressors due to climatic variability could impact amphibians at multiple eco-physiological levels through impacts on endocrine physiology, performance and potentially fitness traits (e.g. reproductive output).

## Introduction

Global atmospheric temperature is warming [1] and extreme thermal events in particular [2] could affect the performance and fitness traits of ectotherms [3]. It is essential to assess the intrinsic physiological systems of ectotherms, especially their reproductive and stress endocrine systems, with exposure to acute thermal stressors. Monitoring these physiological endocrine systems will enable crucial insights into the possible sub-lethal effects of acute thermal stressors on the physiology of amphibians. However, presently there is a huge knowledge gap due to the lack of experimental data showing the physiological endocrine responses of amphibians towards acute thermal stressors. Most recently, [4] demonstrated that exposure to a repetitive and chronic thermal stressor causes permanent elevation of baseline corticosterone and also causes modulation of the physiological stress response to an acute stressor (capture and handling) in the cane toad (*Rhinella marina*). In this study, we aimed to determine whether an acute thermal stressor could also impact the reproductive and stress endocrine systems of the cane toad and causes potential effects on their locomotor performance.

Our study species, the cane toad, is native to Central America and were introduced throughout the Pacific, including Australia, to control sugar cane beetles, however becoming a major invasive species [5]. In Australia, they have become invasive and have spread many thousands of kilometres in the tropics and subtropics and, to a lesser degree, to temperate regions [6]. Climatic modelling [7] forecasts an increase of the range of cane toads in a warmer Australia. Accessibility in numbers and life-history traits (such as exploration of warmer thermal regimes) led us to select the cane toad as the model species. The cane toad is estimated to have a critical thermal maximum (CT_max_) of 42 °C and optimum mean environmental temperature of 25 °C [8]. We designed an experiment in which the corticosterone stress response to an acute stressor (standard capture and handling) was measured in adult male cane toads after being subjected to one of three acute temperature treatments (30°, 35° or 40 °C). We also measured righting responses to infer locomotory performance of the toads after exposure to an acute thermal stressor. Thus, we explored two key questions, (1) whether exposure to an acute thermal stressor increases the corticosterone stress response, and (2) whether exposure to an acute thermal stressor simultaneously suppresses reproductive hormones (e.g. testosterone) as well as reduces performance trait (e.g. righting responses) of the cane toads.

## Methods

### 2.1 Ethics

Animals were caught and examined humanely under a Queensland Government Scientific Purpose Permit (WISP07106310) and the Griffith University Animal Ethics Committee Permit (ENV/08/10/AEC).

### 2.2 Field sampling

Male cane toads [*Rhinella marina* (Linnaeus 1758); total n = 32] were captured from the field between 1900–2100 h in March 2012 (ambient temperature was on average 25°C) in Southport, Queensland, Australia. Body weight (Bwt) and snout ischium length (SIL) of male toads sampled were 65.3+2.4 g and 57.2+2.2 mm respectively. Each male toad was sampled for baseline urine immediately after capture in the wild, which were assayed in the laboratory to determine the levels of urinary corticosterone and testosterone metabolites. This method is used commonly in field and laboratory based conservation endocrinology research [9], [10], [11], [12], [13] and most importantly, urine sampling overcomes the potential of stress being generated by the animals during sampling [14], [15], [16], [17], and this method also provides a reliable index of changes in serum corticosterone and testosterone levels during exposure to acute stressor in cane toads [18], [19]. Urine sampling followed our established field protocols [17], [20], [21], [22], [23]. Toads were kept in individual resealable plastic Ziploc^©^ bags and transferred into the holding facility on the same night (within three hours of field capture).

In the laboratory (an enclosed holding facility with constant temperature set at 25°C), toads were housed individually in plastic containers (15×15×15 cm), termed ‘home containers’, with meshed lids for ventilation over a 14 day acclimation or adjustment period. Overhead fluorescent lights inside the holding facility were operated manually (switched off at 0600 hrs and switched on at 1800 hrs under a reverse light cycle). Urine samples were collected from the toads on days 5, 10 and 14 in captivity to demonstrate that the toads had settled into captivity prior to being subjected to the experimental (thermal heat shock) treatments, this design was based on our previous studies [24], [25].

### 2.3 Acute temperature (heat shock) treatments

After the 14 days of acclimation, toads (n = 8 toads per group, total 4 groups) were subjected to one of three acute temperature treatments (30°, 35° or 40°C) or sampled under a control temperature of 25 °C. On the day of experiment, a baseline urine sample was collected from each toad immediately prior to exposure to the acute temperature treatment. For each toad, this sample represented baseline levels of corticosterone and testosterone metabolites. Experimental temperatures were achieved by using an electric water bath fitted with a thermostat that was switched on exactly one hour earlier to achieve the required temperature. During the experiment, toads were transferred individually into clear buckets (15 cm high × 10 cm diameter) and the buckets were immersed half way into the water bath using clamps. Water could not enter the bucket and the buckets warmed up only through heat transfer from the warmth of the water bath. The toads were exposed to the required temperature (30°, 35° or 40°C) only for 30 min (representing the acute thermal stressor regime) because during preliminary trials we observed that longer exposure periods (over 30 mins) caused heat stroke and death. Toads in the control group were also exposed to the water bath, which was maintained at 25°C for 30 min. After the treatment was completed, all toads were returned immediately into their individual home containers (maintained at 25°C) and subsequent urine sampling was conducted at an interval of 2 h over an 8 h period using the standard capture handling protocol [16]. This sequential urine sampling later (during analysis) enabled us to determine the magnitude of the corticosterone stress responses of the toads after exposure to the acute thermal stressor.

### 2.4 Performance measurement

The righting response score (i.e. the time taken for an individual toad to change its posture from an inverse position to an upright position) was used as an indirect measure of performance [26] during exposure to the different temperatures. Measurements were recorded immediately after removing the toads from the thermal treatment. Each toad was removed from the bucket and placed on its back on the test surface (flat plastic board 15×15×10 mm) and a timer was started immediately. The toad's underbelly was gently pressed by the palm of the experimenter until the hand could be moved away and the toad remained on its back. The latency time (Sec) was the time when the hands could be moved away while the toad remained on its back. The time taken for the toad to upright was recorded as the righting response speed. Urine sampling re-started immediately after completion of the righting response.

### 2.5 Urinary corticosterone and testosterone metabolite enzyme-immunoassays

The urinary corticosterone and testosterone metabolite enzyme-immunoassay (EIA) protocols are described in earlier works [21], [22]. Each EIA used polyclonal antibody (supplied by Coralie Munro, UC Davis, California) that measures the metabolic end-products of corticosterone (mainly glucuronides) or testosterone (combination of glucuronides and sulphates) in amphibian urine. Urinary concentrations of both hormones were standardised to creatinine (Cr) levels to control for water content based on established methods [22]. Urinary testosterone and corticosterone levels were reported as pg hormone metabolites/μg creatinine.

### 2.6 Statistical analysis

All statistical analysis and graphs were done in Prism (Graphpad Software Inc.). All data are presented as mean + S.E. (standard error). All of the data were tested for normality using D'Agostino & Pearson omnibus normality test and where necessary, the hormone data were log_10_ transformed to avoid heteroscedasticity. Probability values of *α*<0.05 were considered to be significant. Preliminarily, we also tested (using repeated measures ANOVA) whether creatinine concentrations in toad urine varied significantly by the thermal treatments.

#### 2.6.1Urinary corticosterone metabolite responses to wild capture and captivity

A One-way repeated measures ANOVA was used to compare mean baseline urinary corticosterone metabolite concentrations between the samples collected in the wild (day 0) and the samples collected during the acclimation period (days 5, 10 and 14). Post-hoc comparisons between time periods (days 0, 5, 10 and 14) were performed using Bonferroni's Multiple Comparison Tests.

#### 2.6.2Effect of acute temperature treatments on urinary corticosterone and testosterone metabolite levels

Log_10_ corticosterone and Log_10_ testosterone metabolite data were analysed separately using two-way repeated measures analysis of variance (ANOVA) with time and treatment temperature (25°, 30°, 35° or 40 °C) as the grouping factors. Comparisons between the treatments (25°, 30°, 35° or 40 °C) for each time period (times 0, 2, 4, 6 and 8 h) were examined with Bonferroni's Multiple Comparison post-test for each steroid.

#### 2.6.3Performance

The mean righting response score (seconds) for toads within each group were compared between groups (control and treatments) using one-way ANOVA followed by post hoc comparisons.

## Results

### 3.1 Urinary corticosterone metabolite responses to wild capture and captivity

**Figure 1:**
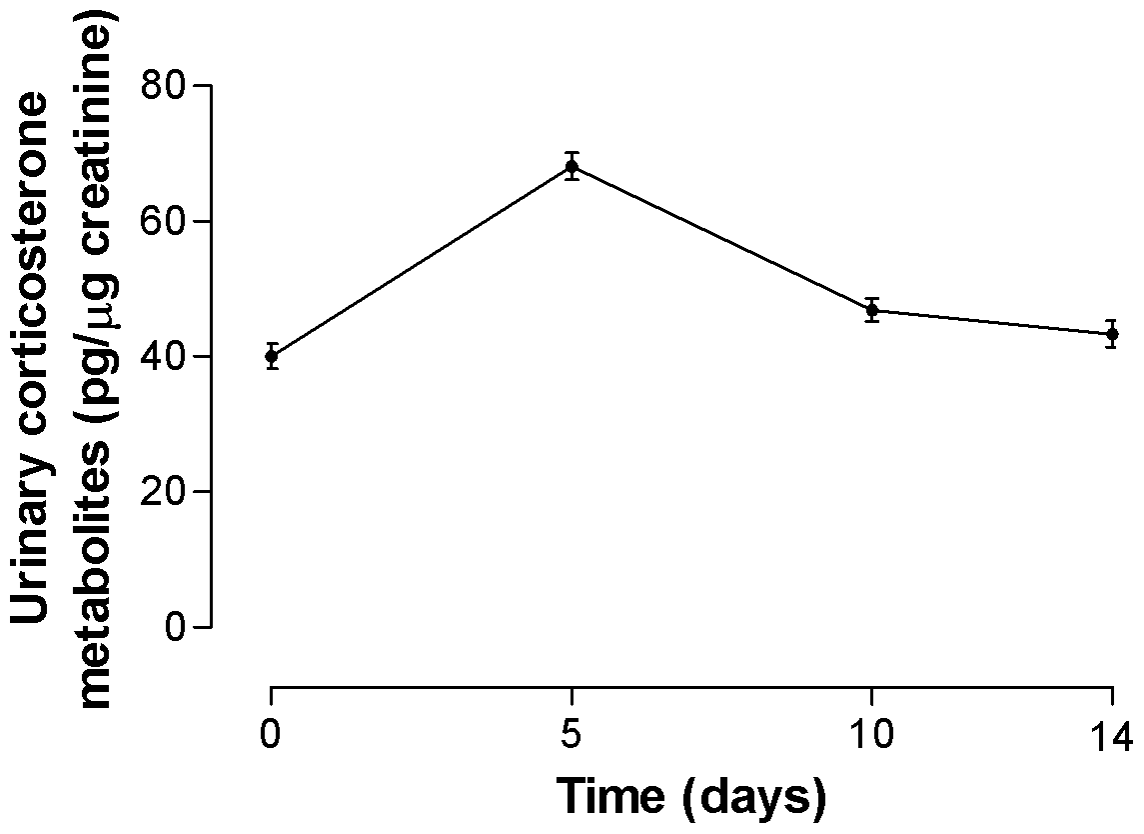
Mean + S.E.M urinary corticosterone metabolite level in male cane toads during 14 days of acclimation at 25°C for 14 days. Sample size for each group was n = 8.

All male toads exhibited urinary corticosterone metabolite responses immediately after being captured in the wild and transferred into captivity (Fig. 1). There was a significant effect of time since initial capture on mean urinary corticosterone metabolite levels (One-way repeated measures ANOVA; F_3, 95_ = 11.69, *p*<0.001). Bonferroni's Multiple Comparison post-test showed no significant difference in mean urinary corticosterone metabolite levels of the toads between day 0 (wild capture) and day 14 (highlighting the completion of the acclimation period).

### 3.2 Effect of acute temperature treatments on urinary corticosterone metabolite levels

**Figure 2:**
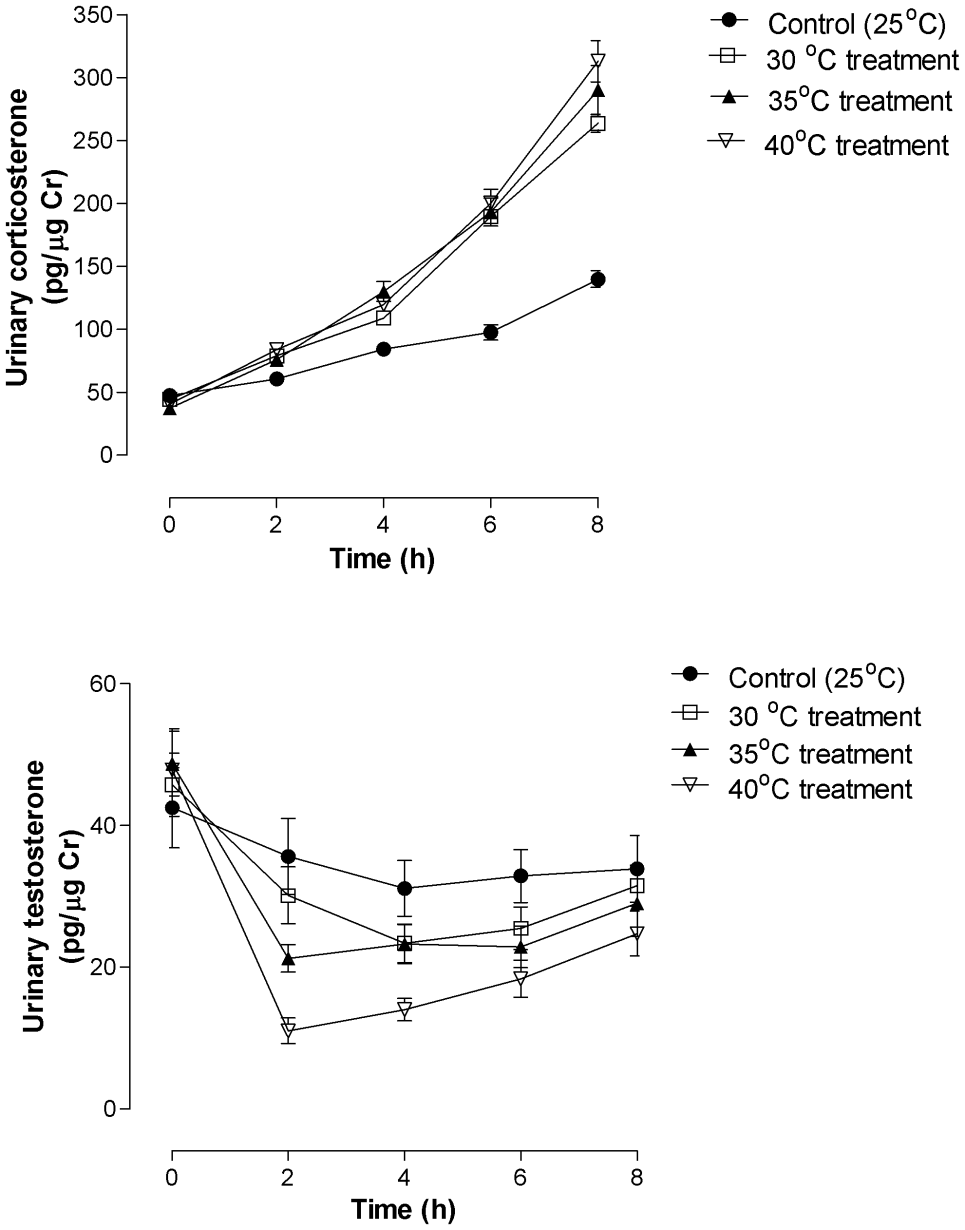
Mean + S.E.M urinary corticosterone (upper panel) and testosterone (lower panel) metabolite responses in male cane toads acclimated at 25°C for 14 days and then subjected to acute temperature treatments of either 30°C (^___^▪^___^), 35°C (^___^▴^___^) or 40°C (- - - ▾ - - -). Control group represents male toads that were sampled using the capture handling protocol at 25°C (- - - • - - -). Sample size for each group was n = 8.

Results showed no significant effect of thermal treatment on urinary creatinine levels that was measured in toad urine samples (urinary levels of Cr ranged from 5–6 μg/ml volume of urine measurement; (Two-way ANOVA F_3, 112_ = 15.25, *p*>0.05). However, there was a significant difference in mean urinary corticosterone metabolite levels between the treatment groups (Two-way ANOVA F_3, 112_ = 35.60, *p*<0.0001) and between times (Two-way ANOVA F_4, 112_ = 558.9, *p*<0.0001), as well as a significant interaction between treatment and time (Two-way ANOVA F_12, 112_ = 22.11, *p*<0.0001). There was no significant difference in mean urinary corticosterone metabolite level between the control group (25°C) and any of temperature treatments (30°, 35° or 40 °C) at time 0 h (prior to exposure of the toads to the water bath; Fig. 2A). However, there was a significant difference in mean urinary corticosterone metabolite level between the control group and all of the temperature treatment groups at times 2, 4, 6 and 8 h post-treatment (Bonferroni posttests *p*<0.05 for all comparisons; Fig. 2A). Mean urinary corticosterone metabolite levels were not significantly different between the three acute temperature treatment groups (0–8 h), except for the 8 h comparison between the 30 °C and 40 °C treatment groups (Bonferroni posttests *p*<0.01; Fig. 2A). Mean urinary corticosterone metabolite levels were higher for the 40°C treatment group in comparison to the 30°C treatment group at 8 h.

### 3.3 Effect of acute temperature treatments on urinary testosterone metabolite levels

Individual urinary testosterone metabolite concentrations of the adult male toads decreased between 0–8 h for all temperature treatments (25°, 30°, 35° or 40 °C; Fig. 2B). The magnitude of this decline was most evident at 2 h, which was for the 40 °C temperature treatment group (Fig. 2B). Mean urinary testosterone metabolite concentrations varied by treatment (Two-way repeated measure ANOVA F_3, 112_ = 4.63, *p*<0.001) and between times (Two-way repeated measure ANOVA F_4, 112_ = 85.18, *p*<0.001), and with a significant interaction between treatment and time (Two-way repeated measure ANOVA F_12, 112_ = 8.96, *p*<0.001). Like urinary corticosterone metabolite levels, mean baseline urinary testosterone metabolite levels were also not significantly different among study groups (Fig. 2B). However, mean urinary testosterone metabolite concentrations for the control group (25°C) were significantly different from the 35 °C and 40 °C temperature treatment groups at 2 h and 4 h post-treatment [Fig. 2B, *p*<0.05] (Fig. 2). However, no significant difference in mean urinary testosterone metabolite concentrations were observed between the control group and any of the temperature treatment groups at times 6 h and 8 h (Fig. 2B, *p*<0.05).

**Figure 3:**
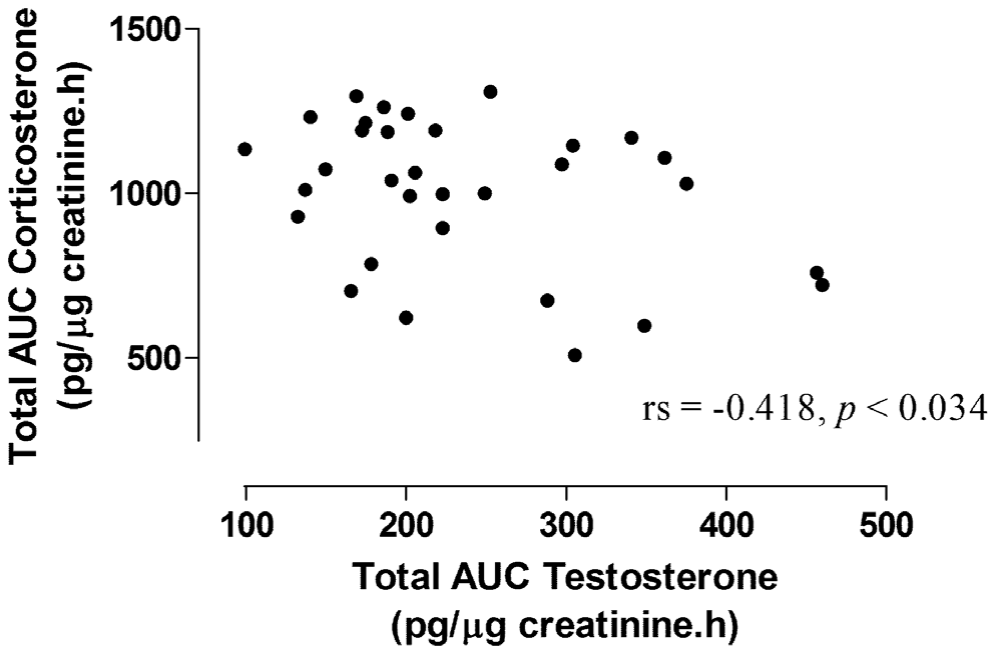
Correlation between total integrated corticosterone and testosterone responses. Temperature treatments are denoted using numbers by the data points.

The results also showed significant negative correlation between urinary corticosterone and urinary testosterone metabolite levels (Fig. 3; r_s_ = −0.458, *p*<0.034, calculated using both steroidal metabolite levels from all four study groups).

### 3.4 Performance

**Figure 4:**
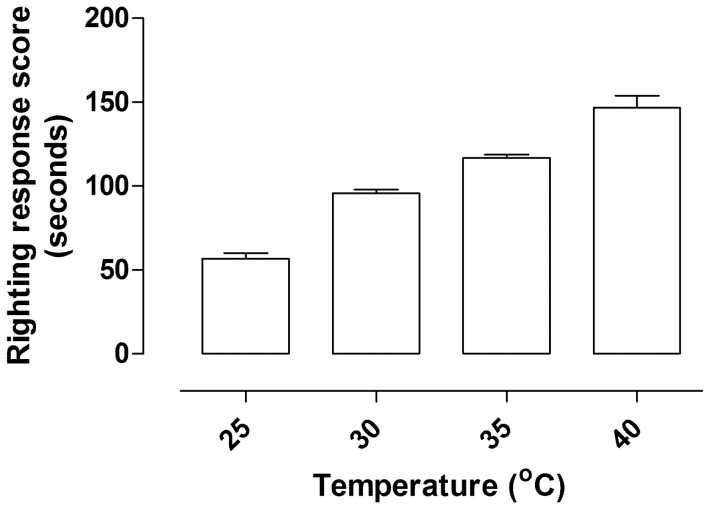
Histogram showing mean durations of righting response (seconds) of male cane toads after exposure to 25°, 30°, 35° or 40°C. Sample sizes at each time point were n = 8.

The mean righting response score of the cane toads differed significantly between the four groups (F _3, 31_ = 78.38, *p*<0.0001, Fig. 4). The toads that were subjected to acute temperature treatment of 45°C had the longest durations of immobility compared to the 30°C and 35°C treatment groups, and control temperature group (Fig. 4).

## Discussion

Our results have demonstrated for the first-time the sub-lethal effects of an acute thermal stressor on the reproductive and stress endocrine axis of an amphibian species, the cane toad. Glucocorticoid responses to a thermal stressor have also been demonstrated in other ectotherms, such as fishes [27], [28]. Earlier, [29] demonstrated *in-vitro* that acute increase in ambient temperature from 5°–30°C led to six fold increase in corticosterone production by the interrenal glands of the marsh frog (*Rana ridibunda*). Furthermore, [30] assessed plasma corticosterone responses to different acute temperature exposures (30 seconds only) in rats showed that exposure to extreme temperature (57 °C) resulted in significant rises in the levels of plasma corticosterone (sampled at 30 minutes post-treatment). In another study, [31] showed that male desert iguanas (*Dipsosaurus dorsalis*) that were maintained at 40 °C for one week exhibited significantly higher plasma corticosterone concentrations in response to an adrenocorticotropic hormone (ACTH) treatment in comparison to individuals that were maintained at 10°, 20° or 30°C.

It is most likely that key biochemical processes, such as metabolic rates of cane toads could be altered under influence of the acute thermal stressor or heat conditions. Therefore, increased secretion of corticosterone under exposure to an acute thermal stressor could be energetically risky because corticosterone is itself a catabolic hormone (increases metabolic rates [32]. Whether increased corticosterone response during exposure to an acute thermal stressor increases or decreases metabolic rates in the toads (to conserve energy) warrants investigation. Our results also showed a reduction in performance (righting responses) during exposure to an acute thermal stressor, Furthermore, our results showed that exposure to an acute thermal stressor also caused reductions in the male reproductive hormone testosterone. It is likely that increased corticosterone response caused a reduction in testosterone levels, as negative associations between the reproductive and stress endocrine axes are well known in amphibians, however this relationship is not universal [20], [33]. Overall, our results suggest that an acute thermal stressor could affect cane toads through multi-level eco-physiological effects by not only increasing physiological stress responses but also by disrupting performance of the toad and reducing reproductive hormones under this experimental situation. Therefore, it will also be worthwhile to investigate whether exposure to acute thermal stressors under natural conditions could also cause ultimate effects on cane toad fitness (e.g. reproductive outputs).

Interestingly, the intensity response curves for testosterone were pretty clear during exposure to the acute thermal stressor, however the intensity response curves for corticosterone were not distinguishable between the thermal treatments until at time period 8 h. Earlier, we have demonstrated that urinary testosterone concentrations in adult male toads (under breeding conditions) were much higher than baseline levels following exposure to a short-term stressor of repeated manual restraint (5 min of manual restraint with hourly intervals of urine sampling) [20]. Therefore, it is plausible that the changes in testosterone and corticosterone depend on the type, duration and nature of the short-term stressor, and also the breeding condition of the study species [25], [33]. Future investigations should take into account the intensity and duration of standard stressor (manual handling) [33] used for assessing short-term corticosterone responses after exposure to particular treatment (e.g. acute thermal stressor). It is recommended that a standard manual stressor (such as 5 minutes of manual restraint) between urine sampling is used so that all toads can be equally subjected to this protocol and the variation in the short-term corticosterone responses could be distinguishable after exposure to the thermal treatment [12], [16]. Furthermore, the current study provides baseline information on the impacts of acute thermal stressor on amphibian endocrine physiology and performance after adaptation to a nominal thermal regime (25°C). Future studies could also explore the impacts of acute thermal stressor on the physiological stress responses, reproductive hormones and performance of amphibians after adaptation to higher temperatures.

As environmental conditions vary in space and time, populations and species are continually challenged to maintain homeostasis and performance [34]. The global climate is changing and increased extreme climatic events [35], [36], [37] can have severe impacts on biodiversity [38], [39], [40]. The significant effects on behaviour, reproductive and stress hormones shown here provide an understanding of the physiological responses of amphibians to extreme thermal stress. Presently, we have no data to show whether acute thermal stressor directly affects the fitness and survival of cane toads, such as through reductions in reproductive outputs. Our experimental design and amphibian model species (cane toad) certainly provides a basis for future investigations into the potential impacts of thermal stressor (especially unpredictable and chronic thermal events) on amphibian physiology, ecology and survival. These investigations will become absolutely necessary for native and more threatened species, particularly those living in hot and arid environments as our study evidences that acute thermal stressors have the capacity to manipulate intrinsic reproductive and stress physiology, and performance of amphibians. Long-term monitoring of physiological stress responses, reproductive hormones and fitness in amphibian species living along climatic gradients (such as mountain endemics threatened with extinction around the world [41], [42]) will benefit from the methods used in this study. Furthermore, studies on physiological tolerance can be used to make predictions about species response to future climatic scenarios, and novel stressors [43], and develop novel strategies to mitigate the imminent extinction crisis.
